# Section 12 approval: fit for purpose?

**DOI:** 10.1192/bjb.2020.38

**Published:** 2020-06

**Authors:** Darryl Ballantyne-Watts

**Affiliations:** Consultant Psychiatrist, Gwent Forensic Psychiatry Service, Aneurin Bevan University Health Board, email: darryl.ballantyne-watts@wales.nhs.uk

I read this article^1^ with some interest, and with some alarm. Yes, striving for ‘evidence-based improvements’ in the Section 12 approval/reapproval process is an understandably good thing. However, basing recommendations on a 21.7% (5/23) return rate for a questionnaire is never going to change much behaviour. Not even when this information is ‘triangulated … with other sources’ are many heads going to be turned.

I believe that those of us who carry out Section 12 assessments in the real world are all too aware of the lack of hospital resources and are thus inclined to seek out every community solution for disposal, given the availability of ‘alternative to hospital’ teams these days. Particularly when we are considering complex mental illness and mental disorder matters in a social context coupled with a healthy assessment of risk, the decision to detain to hospital for assessment cannot be taken easily or lightly.

Knowing the precise wording of mental health law is important, and we all want to ‘do things right’. But in a complex, sometimes heated, community situation we are required to complete the harder additional task of ‘doing the right thing’, which takes time, thought, experience and some element of wisdom. I am not at all sure that the solutions proposed in this paper will take many in that direction.
